# Eradication of methicillin resistant *S. aureus* biofilm by the combined use of fosfomycin and β-chloro-L-alanine

**Published:** 2017-02

**Authors:** Elham Akbari-Ayezloy, Nima Hosseini-Jazani, Saber Yousefi, Nazanin Habibi

**Affiliations:** 1 Students Research Committee, Urmia University of Medical Sciences, Urmia, Iran; 2 Department of Microbiology, Faculty of Medicine, Urmia University of Medical Sciences, Urmia, Iran

**Keywords:** MRSA, Fosfomycin, Phosphomycin, β-chloro-L-alanine, Biofilm, RAPD-PCR

## Abstract

**Background and Objectives:**

Biofilm formation is an important virulence factor for methicillin-resistant *Staphylococcus aureus* (MRSA). Fosfomycin is a borad-spectrum antibiotic with inhibitory effects on biofilm production and β-Chloro-L-alanine (β-CLA) is an amino acid analog. The aim of this study was to determine effect of the combination of fosfomycin and β-CLA on biofilm production by MRSA isolates. Also, the clonal relatedness of the isolates was evaluated.

**Materials and Methods:**

To determine the ability of biofilm production by 42 MRSA isolates, microtiter plate method was used. Antibacterial activities of fosfomycin and β-CLA were investigated by determining MICs and MBCs. Antibiofilm activities were measured in the presence of sub-MIC concentrations of fosfomycin, β-CLA or a combination of both. RAPD-PCR was used for investigating the clonal relationship between isolates by the two specific primers.

**Results:**

21.4% of isolates were strong and 5% were moderate biofilm producers. The effect of fosfomycin plus β-CLA treatment on biofilm production was significantly different from non-treated, fosfomycin and β-CLA groups (p=0.00, 0.004 and 0.000 respectively). RAPD-PCR analysis revealed that the RAPD1 primer had more discriminatory power. The Sizes of RAPD-PCR bands ranged from 150 bp to 1500 bp and the number of bands varied from 1 to 13.

**Conclusion:**

Clonal relatedness of isolates showed that the majority of biofilm producing isolates had identical pattern and only three isolates showed more than 80% similarity. The combination of fosfomycin and β-CLA could be introduced as an excellent mixture for eradication of MRSA biofilms in vitro.

## INTRODUCTION

Methicillin-resistant *S. aureus* (MRSA) is a major public health problem all over the world and infections caused by these microorganisms are among the most important therapeutic challenges. MRSA infections are usually resistant to commonly used antibiotics. In spite of introducing several new antibacterial agents during the past decades, MRSA remains one of the difficult-to-treat ESKAPE pathogens (*Enterococcus faecium, S. aureus, Klebsiella pneumoniae, Acinetobacter baumannii, Pseudomonas aeruginosa,* and *Enterobacter* species). On the other hand, many of the newer antimicrobial agents are associated with adverse side effects, developing resistance issues and high drug costs (1).

Antibiotic resistance and biofilm-forming capacity are very important for the success of *S. aureus* as a pathogen in both healthcare and community settings. Biofilms are populations of multilayered bacterial cells which are growing in an enclosed exopolysaccharide matrix on a surface. *S. aureus* is one of the most common causes of infections related to implanted medical devices. On the other hand, most of the adhered *S. aureus* are MRSA (1, 2).

Fosfomycin (Phosphomycin) is an antibiotic with low molecular weight which inhibits the first stage of peptidoglycan synthesis. It is a broad-spectrum bactericidal antibiotic and is effective against many pathogenic bacteria including MRSA (3).

Fosfomycin shows synergistic effects with some other antibiotics, including glycopeptides, linezolid, betalactams, aminoglycosides and quinolones (4). It can be administered orally, parenterally or locally. It does not bind to plasma proteins and reach high concentrations in the interstitial fluid and tissues. It has been used for treating different kinds of infections in many body sites, including urinary, respiratory, intraabdominal, obstetric-gynecologic, central nervous system and skin infections. It does not cause notable changes in the human microbiota (5).

β-Chloro-L-alanine (β-CLA) is a non-toxic bacteriostatic amino acid analog (6) which inhibits alanin-valine transaminase or transaminase C (MurC) irreversibly. MurC is the enzyme responsible for the assembly of L-alanine to UDP-MurNAC during the intra-cytoplasmic stage of peptidoglycan biosynthesis (7). It seems that two peptidoglycan biosynthesis inhibitory agents have synergistic effects when they are used in combination. Although the antibiofilm effects of fosfomycin in combination with some other antimicrobial agents have been studied before (8), to the best of our knowledge, the effect of β-CLA or its combination with fosfomycin on biofilm production has not been studied so far. Therefore, the aim of the present study was to determine the combined effects of fosfomycin and β-CLA on biofilm production by MRSA clinical isolates. Also, the clonal relatedness of the isolates was evaluated by using two identical primers.

## MATERIALS AND METHODS

### Bacterial isolates and culture media.

*S. aureus* isolates were collected from clinical specimens submitted to the clinical diagnostic laboratories in Urmia, Iran during a six-month period, from July to December 2011. Isolates were further processed by standard phenotypic tests such as colonial morphology, Gram staining, catalase test, ability to grow on mannitol salt agar, DNase (Merck, Germany) and slide as well as tube coagulase tests (9) to identify as *S. aureus.* MRSA was identified by using oxacillin strip test (Hi-media, India) (10). The antibiotic susceptibility pattern of MRSA isolates was determined according to Clinical and Laboratory Standards Institute instructions (CLSI, Kirby Bauer assay). Isolated bacteria were maintained for long storage on skim milk medium (BBL; Becton Dickinson Microbiology Systems Cockeysville, MD21030, U. S. A), by adding 10% glycerol in −80°C. *S. aureus* ATCC25923 was used as the reference strain.

### Analysis of antibiotic susceptibility patterns.

Antibiotic resistance patterns of the isolates against 18 commonly used antimicrobial agents including clindamycin (2 μg), ciprofloxacin (5 μg), erythromycin (15 μg), co-trimoxazole (1.25/23.75μg), nitrofurantoin (300 μg), penicillin G (10 U), rifampin (5 μg), tobramycin (10), tetracycline (30 μg), amikacin (30), gentamicin (10), ceftizoxime (30), ampicillin (10), teicoplanin (30), chloramphenicol (30), co-amoxiclav (20/10), imipenem (10) and cephalotin (30) were determined using the disk diffusion method, according to the CLSI recommendations. All the tested antibiotics were manufactured by the Hi-Media Company, India.

### Biofilm formation assay.

The phenotypic biofilm production was performed by microtiter dish biofilm formation assay. Isolates were grown in TSB for 24 hours at 37°C, then cultures was diluted 1:100 and 200 μL of the prepared inoculum was added to each well of a microplate and incubated overnight at 37°C. According to the protocol (11), eight replicates were tested for each isolate. After incubation, planktonic cells were removed by turning the plate over and shaking out the liquid, then plates were submerged three times in water and shaked out. 200 μL of 0.1% crystal violet solution was added to each well and plates were incubated for 15 minutes at room temperature, then they were submerged in a water tub for 3–4 times, shacked out, knocked on paper towels and dried. 250 μL of acetic acid solution (30% in water) was added to each well to solubilize the dye and plates were incubated at room temperature for 10 minutes. Contents were transferred to a new, flat-bottomed microtiter dish and absorbance was measured by ELISA reader (Awareness technology Inc) at 550 nm.

Biofilm producer isolates was defined as described elsewhere (12). In brief, the optical density cut-off (ODc) value is considered as: average OD of negative control + 3 × SD (standard deviation) of negative control, and the biofilm producers were classified as shown in Table 1.

**Table 1.** Criteria used for the classification of isolates with respect to their biofilm formation

**criteria**	***Biofilm*-formation Capacity**
ODs ≤ODc	No biofilm producer
ODc≤ ODs ≤ 2×ODc	Weak biofilm producer
2 × ODc ≤ ODs ≤ 4 × ODc	Moderate biofilm producer
4 × ODc <ODs	Strong biofilm producer

ODs (average of the optical density of sample).

### Antibacterial activity of fosfomycin and β-chloro-L-alanine:

Antibacterial activities of fosfomycin and β-CLA were carried out by tube-dilution method. In brief, bacterial isolates were cultured overnight at 36±0.5°C on nutrient agar medium, and then suspended in sterile buffer saline and used as inoculate within one hour after adjustment. Bacterial inoculate were added to serial dilutions of fosfomycin (Sigma-Aldrich) or β-CLA(Sigma-Aldrich) containing tubes, with 1.5×10^6^ CFU /mL as the final concentration, by adjusting with No. 0.5 of Mc Farland’s Turbidity Standards. MICs and MBCs of fosfomycin and β-CLA against each isolate were determined by preparing serial dilutions using Mueller Hinton Broth (BBL). Previous studies (9, 13) have reported the concentration ranges for determining MIC and MBC values for fosfomycin and β-CLA to be 0.25–128 mg/L and 0.0625 −1 mM respectively. Experiments were carried out in triplicate. The MIC and MBC values were defined as the lowest antibiotic concentration that completely prevented turbidity in broth or colony growth on agar media after incubation, respectively at 37°C for 24 hours (14).

### Antibiofilm activity of fosfomycin and β-CLA:

Antibiofilm activities of fosfomycin and β-CLA were determined using microtiter dish biofilm formation assay (16). Isolates were incubated in microtiter plates in the presence of sub-MIC concentrations of fosfomycin or 0.5 mM of β-CLA (11). For each isolate eight replicate vials were considered.

### Synergistic antibiofilm activity of fosfomycin with β-CLA.

Sub-MIC concentrations of fosfomycin along with fixed concentrations of β-CLA (0. 5 mM) were added to nutrient broth medium for each isolate and microtiter dish biofilm formation assay method was repeated to measure the amounts of biofilm formation in the presence of both agents.

### RAPD-PCR.

Two identical primers, RAPD7 (GTGGATGCGA) and KAY1 (AGCAGCCTGC), were used to investigate the clonal relationship between isolates by the Random Amplified Polymorphic DNA-PCR (RAPD-PCR) method, as described elsewhere (15). In brief, the genomic DNA of bacterial isolates was extracted using Gram positive DNA extraction kit (SinaClone, Iran). The PCR reaction consisted of: 10× PCR buffer in final concentration of 1× MgCl_2_ (50mM) in a final concentration of 10 mM and dNTP Mix 10 mM in a final concentration of 2 mM; specific primer was added as a final concentration of 10 μM to prepare PCR master mix. PCR amplification was performed in a total volume of 25 μl (23 μl of PCR master mix plus 2 μl of template DNA) in a 0.2 RNase/DNase-free microtubes. PCR amplification program consisted of the following steps: initial denaturation step at 94°C for 4 minutes, followed by 49 cycles repetitions of 60 seconds at 94°C (denaturation), 60 seconds at 28°C (annealing) and 60 seconds at 72°C (extension), with a final extension at 72°C for 7 minutes. PCR products were analyzed by electrophoresis in 1.5% of agarose gel in a TBE buffer at 90 volts. Finally, the gels were stained with safe stain and visualized in gel documentation system and photographed. For each primer the banding pattern was scored as presence (1) or absence (0) of band for each isolate (16). Banding pattern of isolates was analyzed both visually and by online software (http://insilico.ehu.es/dice_upgma/index.php) and dendrogram was built, based on RAPD-PCR results for each primer. The isolates with ≥ 80 similarities in banding pattern were regarded as a single clone.

### Statistical analysis.

The mean optical densities obtained for each isolate in the presence or absence of sub-MIC concentrations of fosfomycin, β-CLA or both were compared and data were statistically analyzed by SPSS 16 software. One-way analysis of variance (ANOVA) was used for analyzing between group means. Differences between means were considered to be statistically significant if the p-value was less than 0.05.

## RESULTS

### Bacterial isolates and culture media.

**Fig. 1.**
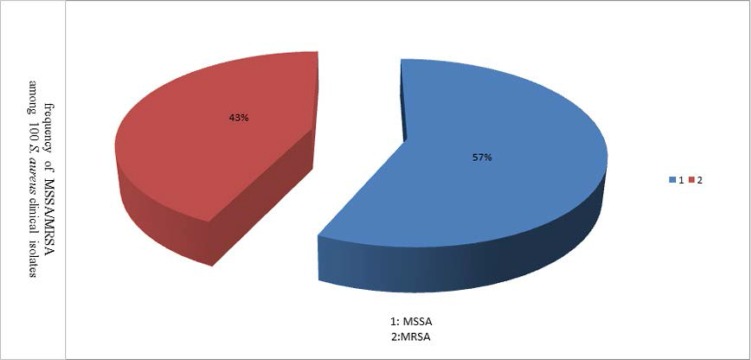
Frequency of MSSA/MRSA among 100 *S. aureus* clinical isolates

One hundred isolates of *S. aureus* were obtained from clinical specimens that were submitted to clinical laboratories during the study. We found no significant differences in frequencies of MRSA infections between genders [22 (51.2%) females versus 21 (48.8%) males]. Of 43 MRSA cases, 35 (81.4%) were isolated from hospitalized patients, and eight from outpatients. The majority of MRSA isolates were cultured from wound (22 cases, 51.2%). 43% of isolates were resistant to oxacillin, so they were defined as MRSA. Because of the lack of enough data about one of the isolates, the study was conducted on 42 MRSA isolates (Fig. 1).

### Analysis of antibiotic resistance patterns.

**Fig. 2.**
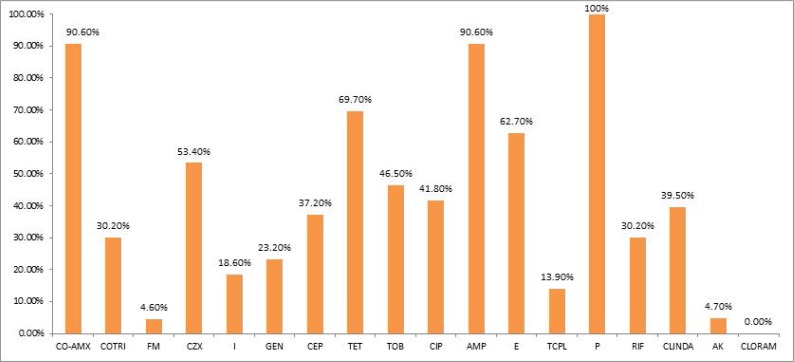
Antibiotic resistance pattern of 43 MRSA isolates CO-AMX(co-amoxiclav), COTRI (co-trimoxazole), FM (nitrofurantoin), CZX (Ceftizoxime), I (imipenem), GEN (gentamicin), CEP (cephalotin), TET (tetracycline), TOB (tobramycin), CIP (ciprofloxacin), AMP (ampicillin), E (erythromycin), TCPL (teicoplanin), P (penicillin G), RIF (rifampin), CLINDA (clindamycin), AK (amikacin), CLORAM (chloramphenicol).

Antibiotic resistance pattern of the 43 MRSA isolates against 18 commonly used antibiotics is shown in Fig. 2. Chloramphenicol, nitrofurantoin, amikacin and teicoplanin were the most active antibiotics against MRSA, respectively. However, the highest amount of resistance was seen against penicillin G. All MRSA isolates were multi- drug resistant.

### Determination of biofilm-producing isolates.

A total of 42 MRSA isolates were investigated with respect to their ability for biofilm formation. In this sense, nine isolates were strong biofilm producers and two isolates were moderate. These isolates were chosen for determining the MIC and MBC of fosfomycin and β-CLA on them. There were no significant differences in the amounts of biofilm formation between isolates obtained from different wards or clinical samples (P>0.05).

### Determination of antibacterial activity of fosfomycin and β-CLA.

The average amount of MIC for all the biofilm producer MRSA isolates was 28.18±14.9μg/mL. Regarding the standard definitions, if the MIC of fosfomycin for each isolate is ≤32 μg/ml, it would be considered as susceptible; so, 45.5% (5 of 11) of isolates were susceptible and the rest were resistant to fosfomycin. However, all the bacterial isolates were resistant to investigated concentrations of β-CLA; hence, for doing further experiments, a defined dose of β-CLA were used according to the previous study (17).

The MIC values and chosen sub-MIC doses for each isolate are shown in Table 2.

**Table 2.** MIC values and sub-MIC doses of fosfomycin against selected isolate

**No. of Isolates**	**MIC(μg/mL)**	**sub-MIC doses(used for determining the antibiofilm effect)**
1	128	64
0	64	32
5	32	16
0	16	8
2	8	4
0	4	2
3	2	1
Total=11		

### Determination of antibiofilm activity of fosfomycin and β-CLAand their combined effects.

Fig. 2 shows the mean optical density± SD for each group in the absence of fosfomycin, presence of sub- MIC doses of fosfomycin, presence of β-CLA (0. 5 mM) or presence of a combination of sub- MIC doses of fosfomycin and β-CLA (0. 5 mM). According to the statistical analysis, the effect of fosfomycin on biofilm production was significant (p=0.017). However, there was no significant difference between the isolates treated with β-CLA and the untreated ones with regard to biofilm formation (p=0.840). Also the effect of fosfomycin+ β-CLA treatment on biofilm production was significantly different from non-treated, fosfomycin and β-CLA groups (p=0.00, 0.004 and 0.000, respectively).

### RAPD-PCR analysis.

RAPD-PCR analysis by two specific primers revealed that the RAPD1 primer has more discriminatory power. Sizes of RAPD-PCR bands ranged from 150 bp to 1500 bp and the number of bands varied from 1 to 13. Isolates with no band were excluded from final analysis (Fig. 4). Clonal relatedness of isolates was evaluated by construction of dendrogram. Fig. 5 showed that the majority of biofilm producing isolates had identical patterns and only three isolates (33.33%) had different patterns, meanwhile showing more than 80% similarity.

**Fig. 3.**
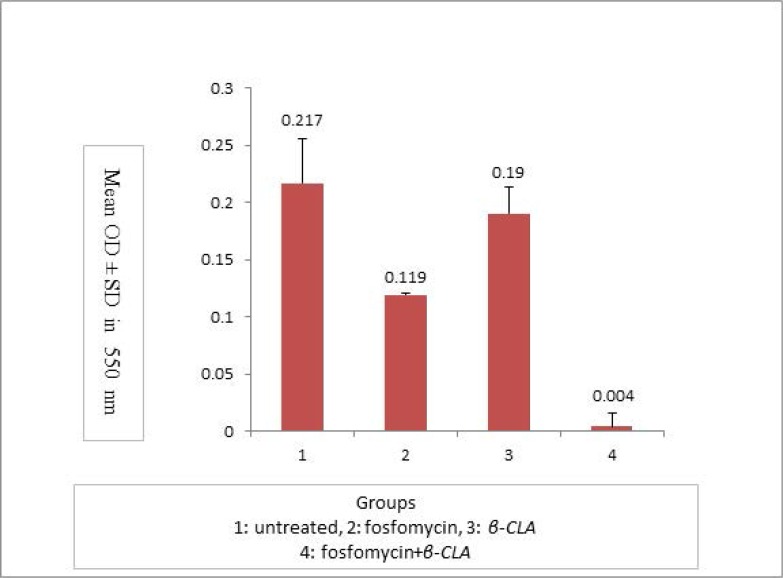
Comprison of biofilm formation in different groups.

**Fig. 4.**
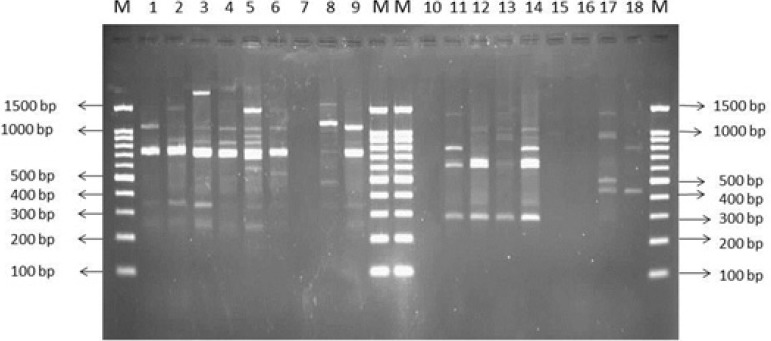
RAPD-PCR banding pattern of MRSA isolates by two specific primers, lane 1 – 9 with RAPD7 primer and lane 10 – 18 with KAY1 primer.

**Table:** 

Lane M: Size marker 100 bp	Lane M: Size marker 100 bp
Lane1: MRSA isolate No. 08	Lane10: MRSA isolate No. 08
Lane2: MRSA isolate No. 17	Lane11: MRSA isolate No. 17
Lane3: MRSA isolate No. 32	Lane12: MRSA isolate No. 32
Lane4: MRSA isolate No. 34	Lane13: MRSA isolate No. 34
Lane5: MRSA isolate No. 88	Lane14: MRSA isolate No. 88
Lane6: MRSA isolate No. 95	Lane15: MRSA isolate No. 95
Lane7: MRSA isolate No. 96	Lane16: MRSA isolate No. 96
Lane8: MRSA isolate No. 97	Lane17: MRSA isolate No. 97
Lane9: MRSA isolate No. 98	Lane18: MRSA isolate No. 98
LaneM: Size marker 100 bp	LaneM: Size marker 100 bp

**Fig. 5.**
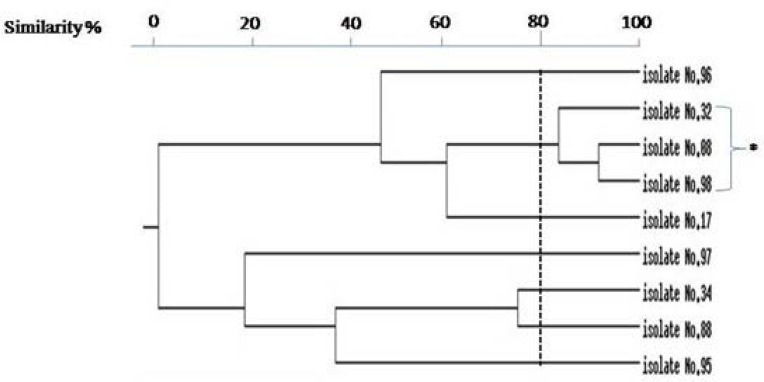
Dendergram of biofilm producing MRSA by UPGMA method. (* Biofilm producing isolates with more than 80% similarity)

## DISCUSSION

Bacterial biofilms can cause major health problems especially in hospital settings such as chronic infections which are difficult to treat and impose longer hospitalization duration and higher treatment expenses. Although MRSA can produce biofilms, it is one of the biofilm producing bacteria which represents high resistance to different classes of antibiotics and is considered as an important threat to the public-health system. In the recent years, different methods have been developed for eradication of biofilms including antibiotics, bacteriophages, and nanoparticles (13).

Tang et al. (2011) studied the efficacy of some antibacterial agents including fosfomycin against biofilm-producing MRSA *in vitro* (17). The mean of fosfomycin MICs against five clinical MRSA isolates was 4.8μg/mL; however, in our study it was notably higher (28.2μg/mL), which in part could be explained by lack of a surveillance system based on epidemiological and molecular techniques in the area of study that prevents emergence, selection and spread of resistant MRSA strains in the community as well as healthcare settings (18).

Plasmid-mediated resistance to fosfomycin is reported by several studies (19); besides, chromosomally resistant mutants were also described (20). These kinds of resistance can develop easily; so, for treating the systemic infections, combination therapies, i.e. including other antibacterial agents in addition to fosfomycin are recommended (17).

Considering all MRSA isolates in our study, disk diffusion test revealed highest amounts of resistance to penicillin (100%), but the least amount of observed resistance was against chloramphenicol, nitrofurantoin, amikacin and teicoplanin, respectively. Similar results have been reported by other researchers from Iran as well as the other countries (21, 22).

In the current study, 11 of 42 (26.2%) MRSA isolates developed biofilm after overnight incubation while some studies reported higher percentages of biofilm producing isolates among MRSA. Cha et al. (23) showed that of the 126 MRSA samples, 86 (68.3%) isolates had biofilm-forming capacity, including five strong level (OD_570_ ≥ 1.0) and 81 weak level (0.2 ≤ OD_570_< 1.0) biofilm producers. Rezaei et al. studied the prevalence of biofilm formation among MRSA isolated from nasal carriers. They showed that all the MRSA isolates have the ability of biofilm formation which 15.4%, 19.2% and 65.4% of them were strong, medium and weak biofilm producers, respectively (24).

However, in our study 81.8% of biofilm producing isolates were strong biofilm producers and only 18.2% of them were moderate in this respect.

Grinholc et al. obtained 302 MRSA isolates and reported that only 45–47% of MRSA were able to produce biofilm *in vitro.* They used RAPD analysis by primer AP-7 (5′GTGGATGCGA3′) to determine clonal structures within the MRSA collection and identified 10 DNA patterns. They found that RAPD has low discriminatory power and they didn’t report any similarity between biofilm forming isolates (25), however in our study the majority of biofilm producing isolates had identical patterns.

Antibiotics commonly used for treating MRSA infections include teicoplanin, vancomycin, rifampin, tigecycline, daptomycin and linezolid (8). In recent years fosfomycin, in combination or alone, is also considered for treating MRSA infections (26). Fosfomycin has a broad antibacterial spectrum and penetrates well into different body sites and crosses the blood–brain barrier (27), so it is an excellent choice for treating deep-seated infections such as diabetic foot infections (28). In addition to its antibacterial activity, fosfomycin exerts immunomodulatory effects, mainly on T-lymphocytes, B-cell and neutrophils (4). Biofilm infections, including catheter and implant associated infections, affect many people and are among the main groups of causative agents of death all over the world (39). Biofilm producing bacteria can cause untreatable chronic infections due to the multicellular composition of biofilms and resistance or tolerance of bacterial cells in biofilms to antibacterial agents and immune responses (30). Therefore, high concentrations of antimicrobials is recommended for eradication of such infections and different kinds of side effects are associated with the usage of high doses of antibiotics (31). There are some reports about the adverse effects of fosfomycin usage in 1–10% of patients including nausea, vaginitis, rash, headache, rhinitis and rarely pseudomembranous colitis (32).

β-CLA is the inhibitor of MurC enzyme in a way which interferes with the L-alanine during peptidoglycan biosynthesis (33). On the other hand, it has been shown that β-CLA, as one of the inhibitors of early stages of peptidoglycan biosynthesis, can strongly reduce methicillin resistance in MRSA (6). Also, there are some reports about bacteriostatic effects of β-CLA on some bacteria including *Salmonella typhimurium* which acts on this bacterium as an effective growth inhibitor (34). The dose-dependent effects of some D-amino acids (including D-methionine, D-phenylalanine and D-tryptophan) on enhancement of the activity of antimicrobials (clindamycin, rifampin and vancomycin) against biofilms of MRSA have been shown elsewhere (35). However the synergism of fosfomycin and β-CLA in reduction of biofilm amounts produced by MRSA clinical isolates has not been investigated so far.

To the best of our knowledge, the present study is the first study investigating the inhibitory effects of the combination of fosfomycin and β-CLA on biofilm production. The results of the present study showed that the highest reduction in MRSA biofilm production in microplate wells was achieved with fosfomycin used in combination with β-CLA (98.2%), which was superior to fosfomycin (45.1%) or β-CLA (12.4%) alone. These results suggest that β-CLA may be an excellent combination partner for fosfomycin but can not replace fosfomycin in eradication of MRSA biofilms.

However, there are several limitations to our study that should be noted. We investigated only the in vitro model of biofilm formation on microtiter plates made of Polystyrene. As different results may be obtained with other types of materials, further investigations are necessary in order to imitate biofilm production by MRSA on the surface of materials used in medical devices and prostheses, such as plastics, steel, teflon, or titanium as well as in vivo studies. Additionally, the duration of biofilm production in our study was limited to overnight incubation; better results may be obtained in a longer experimental period.

In conclusion, the results of this study indicated the ability of 26.2% of MRSA clinical isolates in biofilm production. Biofilm formation significantly decreased in the presence of the combination of β-CLA and fosfomycin (p=0.00); so, it could be introduced as an excellent mixture for eradication of MRSA biofilms *in vitro.* Meanwhile more studies are advised on real models of biofilm formation by this bacterium.
